# Intensive naming training for low-educated demented and non-demented elderly

**DOI:** 10.1590/1980-57642020dn14-040011

**Published:** 2020-12

**Authors:** Amanda Cristina de Souza Ferreira, Ariely Aurélio Silva, Lorrane Rodrigues Paiva, Corina Satler, Maysa Luchesi Cera

**Affiliations:** 1 Faculdade de Ceilândia, Universidade de Brasília – Brasília, DF, Brazil.

**Keywords:** aged, dementia, aphasia, anomia, language therapy, language, idoso, demência, afasia, anomia, terapia da linguagem, idioma

## Abstract

Complaints about naming difficulties may be common in the elderly. In dementia, anomia is the most frequent symptom of language disorders. Naming training can improve lexical access and promote better quality of communication for elderly with or without dementia.

**Objective:**

To analyze naming scores, response time and the generalization of responses for naming of neurotypical and demented low-educated older adults before and after receiving a naming training program, with and without oral comprehension stimulation.

**Method:**

Twenty elderly participants, 10 with dementia and 10 neurotypical, were included after interview, screening for cognition and functionality. The naming training was based on retrieval practice and carried out in 5 sessions. Half of the group underwent exclusive naming training, while the other half received naming training associated with oral comprehension stimulation.

**Results:**

Elderly people with dementia performed better after training for scores on oral naming and comprehension of oral words, except for object manipulation. The response time for naming trained and untrained stimuli was also better for elderly people with dementia. After the intervention, neurotypical individuals performed statistically better in comprehension time and in the score in oral naming, comprehension of oral words and object manipulation, for trained and untrained words.

**Conclusion:**

Naming training, exclusive or associated with oral comprehension, using the recovery technique benefits the language performance of neurotypical and demented elderly, and provides improvements even for untrained stimuli.

## RESUMO.

A queixa de dificuldades para nomear pode ser comum em idosos. Na demência, a anomia é a manifestação mais frequente dos transtornos de linguagem. O treino de nomeação beneficia o acesso lexical e promove maior qualidade de comunicação para idosos com ou sem demência.

**Objetivo:**

Analisar as respostas de nomeação, o tempo de resposta e a generalização das respostas de nomeação de idosos neurotípicos e com demência de baixa escolaridade antes e após um treinamento de nomeação, com e sem estimulação da compreensão oral.

**Método:**

Participaram deste estudo 20 idosos, 10 com demência e 10 neurotípicos, incluídos após entrevista, rastreio de cognição e funcionalidade. O treinamento de nomeação foi baseado na prática de recuperação e realizado em cinco sessões. Metade do grupo foi submetido ao treino de nomeação exclusivo e metade ao treinamento associado à estimulação de compreensão oral.

**Resultado:**

Idosos com demência apresentaram melhor desempenho após o treinamento para pontuações na nomeação oral e compreensão de palavras orais, exceto para manipulação de objetos. O tempo de resposta para nomear estímulos treinados e não treinados também foi melhor para idosos com demência. Após a intervenção, os indivíduos neurotípicos tiveram desempenho estatisticamente melhor no tempo de compreensão e no escore na nomeação oral, compreensão de palavras orais e manipulação de objetos, para palavras treinadas e não treinadas.

**Conclusão:**

O treino de nomeação, exclusivo ou associado ao de compreensão oral, por meio da técnica de recuperação, beneficia o desempenho de linguagem de idosos neurotípicos e com demência e proporciona melhoras inclusive para estímulos não treinados.

## INTRODUCTION

By 2050, Brazil will have over 66.5 million individuals aged over 60.^1^ Dementia prevalence in the country exceeds the global rate and appears to be associated with a longer stage of late life during which individuals present disability.^2^ According to the Diagnostic and Statistical Manual of Mental Disorders (DSM-5), dementia is a disorder affecting one or more cognitive domains, such as complex attention, executive function, learning, memory, language, motor perception and social cognition.^3^ In 2015, the disorder affected an estimated of 5% of elderly worldwide, equivalent to 47 million people, a figure set to rise to 132 million by 2050.^4^ Only in 2018, the total estimated cost of dementia was 1 trillion dollars, an amount which could double by 2030.^5^

Language disorders, such as aphasia, can be early markers of dementia.^6^ Aphasia affects communication and can lead to social isolation and loss of autonomy.^7^ A common language problem in dementia is anomia, which can be caused by multifactorial impairment involving multiple cortical regions.^8^ Dementia due to Alzheimer's disease (AD) is associated with semantic,^9^ morfosintactic^9^ and phonetic-phonological changes,^10^ with failures in emission^9^ ^,^ ^10^ and understanding.^11^ On the other hand, among the language disorders described in vascular dementia (VD), individuals can often have trouble in finding the right word for objects, naming, comprehension, and the presence of incomprehensible speech.^12^

Cognitive training can help minimize the effects of cognitive impairments of dementia,^13^ in which semantic and phonological techniques can improve word retrieval.^14^ For anomia, interventions seek to improve naming through association among information units to access mental lexical representations at lexicosemantic and phonological levels.^15^ ^,^ ^16^ Intensive language therapy for cases of chronic aphasia have produced positive results and generalization of responses.^17^ Retrieval practice is a therapeutic method used for training naming.^15^ ^,^ ^18^ In the technique, the object and its name are first presented for association. Subsequently, each object is presented after a predetermined time interval, for retrieval of the target name from long-term memory by the individual.^19^ Therapeutic interventions aimed at training a specific ability are effective.^19^ Cognitive psychology suggests that intensive training can aid learning of a specific ability.^20^

The majority of studies on language training in dementia have focused on naming rehabilitation. No studies on interventions training comprehension in demented individuals were found in the literature searched. With regard to cognitively healthy older adults, gains in language comprehension after working memory training have been reported, with improvements persisting at 6-month follow-up.^21^

Performance of elderly with memory complaints may not necessarily be associated with the results of cognitive assessments, but with depressive symptoms.^22^ Moreover, these complaints might be attributable to mild cognitive impairments not detected on objective assessments.^23^ Age is a known risk factor for dementia, but other variables have been found to be relevant for reducing the risk of the disease.^24^ Cognitive activities performed throughout the life span, such as formal study, are recognized modifiable factors for linguistic-cognitive performance.^24^ ^,^ ^25^ Thus, controlling modifiable risk factors in dementia is vital, underscoring the importance of cognitive training, including for elderly without dementia.

Elderly submitted to specific training, compared to those who were not, showed gains from cognitive training in executive functions, such as processing speed.^26^ It is noteworthy that processing speed, among other characteristics, can be associated with degree of task complexity or be attributed to a non-cognitive sensory-motor factor and should not be confounded with reaction time on tests.^27^ Although naming ability changes in aging, slowing reaction time for naming tasks can be seen in elderly.^28^

Regarding naming training for typical adults, retrieval practice benefits comprehension and production for foreign language learning.^18^

Given the growing elderly population, the dementia prevalence, the major economic and social impact of the disease, as well as the damage caused by language impairments, it is important to understand the benefits of naming training for elderly people with and without dementia. It is also useful to analyze the impact of naming, exclusive or associated with comprehension stimulation, based on retrieval practice in the language performance of Brazilian natives with low education.

Considering the language changes in AD and VD described above, as well as the interference of schooling in the performance of language tasks, the study hypothesis is that the training provides benefits for elderly people with and without dementia, in terms of naming scores, response time and oral comprehension tasks. It is also expected that naming training associated with oral comprehension training will offer greater language improvements in relation to the exclusive naming training.

Therefore, the objective of the present was to analyze naming scores, response time and the generalization of responses for naming in neurotypical and demented low-educated older adults receiving a naming training program, with and without oral comprehension stimulation.

## METHOD

The study was approved by the Research Ethics Committee, under CAAE number 99251118.6.0000.8093 (document 3.061.928). All participants signed a free and informed consent form, drawn up in conformance with resolution CNS 466/2012.

The sample comprised 20 individuals aged >60 years, comprising 10 neurologically normal and 10 demented low-educated subjects. Five subjects from each group received exclusive naming training, while the other 10 received naming training associated with oral comprehension training.

Written, informed consent was obtained from all participants and their caregivers (when appropriate). The demented individuals were diagnosed and referred to geriatricians within the public health system of the Ceilândia region of the Distrito Federal. The inclusion criteria for the demented elderly were: accompanied by a caregiver; aged >60 years; clinically diagnosed with probable dementia due to AD^29^ or VD;^30^ and dependent for instrumental activities of daily living (IADLs) on the Lawton and Brody Index.^31^

The neurologically normal elderly were recruited by convenience from the same region and matched with the demented patients for sex, age, and educational level. The criteria for inclusion of the healthy elderly were independence for IADLs and a global cognitive score exceeding the normative value for the Brazilian population.^32^

The exclusion criteria for participants were: prior history of alcohol and/or drugs use; uncorrected hearing or visual loss which hampered performance of the tasks proposed in this study; having Portuguese as their second language; diagnosed with neurodegenerative diseases other than Alzheimer's or VD; history of any previous psychiatric diseases; and use of benzodiazepines or other medications that could affect linguistic-cognitive performance.

### Data collection instruments

The participants attended a session prior to the training in which the following assessments were carried out:

- Mini-Mental State Exam (MMSE):^33^ for brief screening of performance on cognitive functions;
- IADL assessment scale:^31^ to assess independence of the individual for performing IADLs;
- Oral naming, oral word comprehension and object manipulation subtests from the Montreal Toulouse Language Assessment Battery (MTL Battery):^34^ for assessing trained language abilities. Each subgroup of participants received a naming training, with and without oral comprehension stimulation. Besides score, time to response onset was also calculated for each item. On the comprehension test, the chronometer was started from the time at which the participant had visual access to the image and audio, and was stopped when the participant showed first signs of responding, such as starting to move a limb. For naming, the chronometer was started from the point at which the participant had visual access to the image and audio and was stopped when the participant began moving orofacial structures. Response time was determined only for correct answers and the mean of correct answers for the skill assessed was calculated.

In addition, all participants underwent a naming assessment comprising trained and untrained items (Supplementary Materials, Chart 1), as outlined below:

- naming of trained items: 10 words used in the naming training were selected for assessment;
- naming of untrained items: 10 words semantically and phonologically matched with 10 trained stimuli were used for assessment.

Results for naming of trained and untrained items were recorded for number of right answers and average time elapsed between presentation of stimulus and start of response by the participant. After training, all participants were reassessed using the following stimuli: the subtests from the MTL Battery applied before the training, and naming of trained and untrained stimuli.

The assessment and training sessions took place at participants’ homes, in a place with less noise or interference, and the order of presentation of the tests and training stimuli was the same for all participants.

### Training

On the same day, after the initial assessment, training was started for five consecutive days, with reevaluation on the fifth day. The two training interventions, i.e. the exclusive naming training and naming training associated with oral comprehension, consisted of sessions of around 30 minutes each. All participants were exposed to stimuli, presented in the same manner on a notebook using PowerPoint software.

Naming training was an adapted version of the retrieval practice method.^18^ The stimuli with images and sound recordings of the corresponding names were first presented to reinforce the association between meaning and word. After this stage, the images were presented again, but sound recordings were available only after 3 seconds. Individuals were asked to name the image upon display, before the sound recording was played. Each trained stimuli was presented only once on each training day.

Naming training consisted of 40 nouns divided into 4 blocks of the following categories: parts of the body, food/foods, animals and household objects (Supplementary Materials, Chart 2). Semantic category was the main variable considered in word selection, since the semantic field is less vulnerable to the effects of education than the phonological field.^35^ The semantic class of the animals category was selected based on a Brazilian study which deemed this category low difficulty for the verbal fluency test for Portuguese,^36^ whereas the other items were chosen for their occurrence in everyday routine activities. As phonological criteria, all of the words of the blocks were selected according to number of syllables and phonemes. Each word was sound recorded and had an average duration of 1 second. An image was selected from the internet for each noun and these were standardized for size to 400×400 and placed against a black background. The stimuli were presented in the same order.

For participants of the oral comprehension training, 4 images were presented at the same time for 4 seconds (Supplementary Materials, Chart 3), an auditory stimulus was then played from a sound recording. Participants were given a further 4 seconds to point out the image that matched the word heard. The stimuli were 20 out of the 40 nouns from the exclusive naming training, and the same phonological control was adopted.

### Statistical analysis

The results of the assessments were tabulated in a spreadsheet and descriptive and inference analyses carried out. The dependent variables were: score and response time on the language assessment. The independent variables were: assessment timepoint, before or after training; study group, with or without dementia; type of naming stimulation, with or without comprehension training; and item type, trained or untrained stimuli.

The Shapiro-Wilk test was applied to determine the distribution of the data and define selection of non-parametric tests.

Wilcoxon's test was used for comparisons pre and post-training and also for comparing performance on trained versus untrained stimuli. The Mann-Whitney test was used to compare after-training performance of demented versus non-demented groups and between the two training types.

A p-value of 0.05 was adopted to indicate statistical significance. All data were analyzed using the IBM Statistical Package for the Social Sciences (SPSS), version 22, software program.

## RESULTS

The sociodemographic data and scores on cognitive screening tests and IADL assessment are given in Table 1. Overall, group participants were 60% female and had a mean education of 2–3 years.

**Table 1:** Demographic and clinical data of demented and neurotypical elderly that participated in one of the two types of naming training interventions

		Demented elderly		Neurotypical elderly	
		Comprehension plus naming training	Naming training alone	Comprehension plus naming training	Naming training alone
		(n=05)	(n=05)	(n=05)	(n=05)
Gender (%)	Female	60	60	60	60
	Male	40	40	40	40
Age – mean (SD)		69.60 (5.46)	79.20 (8.11)	68.80 (4.97)	78.00 (7.58)
Education (years) – mean (SD)		2.60 (2.41)	2.00 (2.00)	2.80 (1.79)	2.20 (1.48)
MMSE – mean (SD)		19.40 (3.85)	15.40 (4.67)	25.00 (3.81)	24.60 (3.36)
LBI – mean (SD)		10.60 (7.33)	17.00 (10.90)	0.20 (0.45)	0.60 (0.89)

SD: standard deviation; MMSE: Mini-Mental State Exam; LBI: Brody & Lawton Index.

The analysis of differences in language performance before and after training for each group using the Wilcoxon test (Table 2) revealed that the demented elderly had a statistically significant difference between the two assessment timepoints. Results showed improved post-training performance for scores on the MTL Battery subtests, except for object manipulation and response time scores for naming trained and untrained stimuli.

**Table 2:** Language performance before and after naming training, by group studied.

Subtest	Demented elderly			Neurotypical elderly		
	Assessment timepoint			Assessment timepoint		
	Before	After	p-value	Before	After	p-value
	Mean (SD)	Mean (SD)		Mean (SD)	Mean (SD)	
MTL – Comprehension (number of correct words)	3.60 (0.70)	4.40 (0.70)	0.011	4.90 (0.32)	5.00 (0.00)	0.317
MTL – Comprehension (time/sec)	4.88 (1.45)	3.84 (1.96)	0.059	2.08 (0.41)	1.75 (0.35)	0.005*
MTL – Object manipulation (number of correct words)	13.00 (3.43)	14.10 (2.28)	0.121	15.90 (0.32)	15.90 (0.32)	1.000
MTL – Object manipulation (time/sec)	3.59 (2.04)	4.07 (1.52)	0.721	1.99 (0.34)	1.71 (0.35)	0.059
MTL – Naming (number of correct words)	15.30 (6.32)	17.50 (5.19)	0.007*	26.00 (3.43)	29.30 (1.16)	0.011*
MTL – Naming (time/sec)	6.02 (1.69)	5.60 (1.31)	0.114	2.11 (0.62)	1.94 (0.34)	0.262
Trained words (number of correct words)	5.80 (2.10)	8.90 (1.10)	0.007*	8.40 (1.17)	10.00 (0.00)	0.010*
Trained words (time/sec)	5.69 (1.87)	2.37 (1.03)	0.005*	1.78 (0.23)	1.57 (0.18)	0.059
Untrained words (number of correct words)	5.60 (2.63)	7.10 (1.85)	0.010*	8.30 (1.06)	9.70 (0.48)	0.006*
Untrained words (time/sec)	5.77 (2.36)	4.55 (2.10)	0.022*	2.00 (0.23)	1.90 (0.34)	0.284

SD: standard deviation; MTL: Montreal Toulouse Language Assessment Battery; Wilcoxon's test;

* p<0.050.

The non-demented individuals had statistically better performance for comprehension time on the MTL Battery subtest and for score on the three naming measures from the MTL Battery for trained and untrained words. It is worth mentioning that the elderly without dementia had scores within the normative data on the MTL Battery for their age group and education.

Comparison of language performance of elderly with and without dementia revealed a statistically improved performance of demented elderly on scores for the oral comprehension and object manipulation tests of the MTL Battery and for naming time of untrained words (Table 3).

**Table 3:** Comparison of demented and neurotypical elderly for language performance after training.

	Demented elderly	Neurotypical elderly	p-value
	Mean (SD)	Mean (SD)	
MTL – Comprehension (number of correct words)	0..80 (0..63)	0..10 (0..32)	0..007*
MTL – Comprehension (time/sec)	-0..32 (1..31)	0..32 (0..21)	0..059
MTL – Object manipulation (number of correct words)	1..40 (2..01)	0..00 (0..00)	0..005*
MTL – Object manipulation (time/sec)	-0..55 (1..62)	0..28 (0..45)	0..257
MTL – Naming (number of correct words)	2..20 (1..93)	3..30 (3..06)	0..396
MTL – Naming (time/sec)	0..54 (1..42)	0..17 (0..37)	0..597
Trained words (number of correct words)	3..10 (2..08)	1..60 (1..17)	0..085
Trained words (time/sec)	1..21 (1..25)	0..20 (0..26)	0..005*
Untrained words (number of correct words)	1..50 (1..18)	1..40 (0..84)	0..904
Untrained words (time/sec)	-0..22 (1..01)	0..10 (0..37)	0..290

SD: standard deviation; MTL: Montreal Toulouse Language Assessment Battery; Mann-Whitney's U test;

* p<0.050.

Comparison of language performance for the two types of naming training, with and without oral comprehension stimuli, revealed a statically significant difference only for naming time of stimuli from the MTL Battery, with better response for naming training only (Table 4). Comparison of performance between naming of trained and untrained words after intervention showed a statistically significant difference in score and naming time (Table 5). The scores for naming trained words were statistically higher than for untrained words, while naming time was statistically shorter.

**Table 4:** Comparison of language performance for training interventions.

Assessment subtest	Training type		p-value
	Naming plus oral comprehension training	Naming training alone	
	Mean (SD)	Mean (SD)	
MTL – Comprehension (number of correct words)	0.50 (0.53)	0.40 (0.70)	0.511
MTL – Comprehension (time/sec)	0.05 (1.02)	-0.05 (0.97)	0.650
MTL – Object manipulation (number of correct words)	0.30 (0.48)	1.10 (2.13)	0.779
MTL – Object manipulation (time/sec)	-0.39 (1.60)	0.12 (0.72)	0.406
MTL – Naming (number of correct words)	2.20 (2.15)	3.30 (2.91)	0.396
MTL – Naming (time/sec)	-0.16 (0.65)	0.87 (1.10)	0.016*
Trained words (number of correct words)	2.20 (1.87)	2.50 (1.84)	0.505
Trained words (time/sec)	0.50 (0.46)	0.91 (1.38)	0.910
Untrained words (number of correct words)	1.50 (1.27)	-0.30 (0.68)	0.873
Untrained words (time/sec)	1.40 (0.70)	0.18 (0.79)	0.112

SD: standard deviation; MTL: Montreal Toulouse Language Assessment Battery; Mann-Whitney's U test;

* p<0.050.

For this analysis. the difference was calculated by subtracting results for previous assessment from those of subsequent assessment.

**Table 5:** Comparison of naming responses of trained and untrained words after training.

	Trained words*	Untrained words*	p-value
	Mean (SD)	Mean (SD)	
Total correct words	9.45 (0.94)	8.40 (1.87)	0.005*
Time (sec)	1.50 (0.31)	2.37 (0.88)	<0.001*
Words – Difference in value before and after training	2.35 (1.81)	1.45 (1.00)	0.018*
Time (sec) – Difference in value before and after training	0.71 (1.02)	-0.06 (0.76)	0.025*

SD: standard deviation; Wilcoxon's test;

* p<0.050.

For this analysis. the difference was calculated by taking the result after training and subtracting the result before training.

## DISCUSSION

The main results obtained were related to the improvement in naming and oral comprehension performance of demented and non-demented elderly in language assessments after a training intervention: naming training program, with and without oral comprehension stimulation. The demented elderly showed greater improvement on the language measure than the neurotypical elderly. The two types of training promoted oral language performance improvements, with greater values for the exclusive naming training for response time to stimuli trained. In addition, the responses for the trained words were statistically better than for untrained words, although benefit on performance were evident for both types of stimuli.

The sample included elderly with a low level of education averaging two years of study.

Previous studies have associated schooling with performance in cognitive language tests, such as naming^37^ ^,^ ^38^ and verbal fluency tests.^39^ Meantime, an study have shown that low educational level allows for greater therapeutic gains,^40^ despite being higher risk factors for dementia^24^ and associated with worse scores on language testing.^25^ However, according to Clark et al.,^40^ the effects of cognitive training reduce educational disparities in risk of dementia.

Regarding comparison of language performance before and after a naming training, the two groups of elderly showed improved mean scores and response time post-training, i.e. there were language benefits in terms of response time on language assessments, as well as scores, corroborating the benefits of cognitive training for elderly.^40^ ^,^ ^41^

The results of this study demonstrated that individuals with dementia, as well as neurotypical elderly, showed benefits when using semantically paired tasks of comprehension and naming. Our findings are in agreement with previous studies that have reported that the use of semantic categories in comprehension training may be associated with a benefit for aphasic naming,^42^ and that non-semantic tasks are not effective in recovering the word.^43^

Comparison of training benefits for language performance of elderly with and without dementia revealed a statistically improved performance of demented elderly in oral comprehension test scores and response time for naming untrained stimuli. Although lower, the results for neurologically healthy elderly also showed a statistically significant improvement. The healthy elderly group had better language performance on baseline assessment, exhibiting few language errors. While higher educational level is associated with better performance on language tests,^25^ lower education allows for greater potential therapeutic gains.^40^ The present study demonstrated the benefits of naming training, particularly for older adults who had previously performed poorly on language tests. These results corroborate the findings of the study by López-Higes et al.,^44^ who also found that cognitive training has a more positive outcome in patients with lower cognitive reserve than in patients with higher cognitive reserve. Thus, our results are in line with the alternative approach considering that participants who start the intervention with a lower initial level of trained function finish with better post-training results.^45^

Comparison of language performance for the two types of naming training, with and without oral comprehension stimuli, revealed a statistically significant difference only for naming time of stimuli from the MTL Battery, with better response for exclusive naming training. Response time was not a requirement in instructions for the assessment tests, although response time was measured. Reaction time is less associated with scores on tests that rely on speed than with untimed tests.^27^ Thus, it is likely that tests with instructions requesting a faster response from participants could better identify training effects. It is therefore suggested a reorganization of language processing after training, particularly in specific naming training.

The benefits of the training, including the comparison pre-and post-training in naming of untrained stimuli, are in line with a study on intensive language therapy, despite methodological differences.^17^ As expected, scores for naming trained words were statistically higher than for untrained words. Naming time was statistically faster, showing improved performance for the stimuli trained.

Results showed that naming training, with and without oral comprehension stimulation, favored shorter response time with an increased in the number of correct answers in naming by Brazilian elderly with and without dementia. The exclusive naming training showed best results, and demented elderly improved the most. After training, there was generalization of improvement in naming for the untrained stimuli.

Future studies should attempt to adapt the training without technological resources to facilitate access for people with low education who may also not have access to technology and facility with its manipulation.

The present study has the following limitations:

- The small sample showed improvement on performance after training, but results could be even greater for larger samples;
- The sample included elderly from a single region of Brazil: studies involving other regions would be valuable, given that demographic variables influence cognitive performance;
- The benefit of training among the elderly with higher educational level or subdividing the types of dementia was not explored;
- Maintaining long-term performance improvement and the possible learning effect were not investigated;
- Training sessions were held at participants’ homes, a setting with visual and auditory interference which may have affected answers. These variables were controlled for by providing guidance on setting up the best environment possible;
- Response times were recorded manually using a cell phone device. Electronic timers could minimize the motor and attention effects of the examiner during manual recording;
- The perceived benefits of the intervention, although reported by some of the participants, were not formally assessed.

## References

[1] Instituto Brasileiro de Geografia e Estatística (2018). Projeções e estimativas da população do Brasil e das Unidades da Federação: Pirâmide etária 2018-2058.

[2] Burlá C, Camarano AA, Kanso S, Fernandes D, Nunes R (2013). A perspective overview of dementia in Brazil: a demographic approach. Ciênc Saúde Coletiva.

[3] American Psychiatric Association (2014). DSM-5: Manual diagnóstico e estatístico de transtornos mentais. Transtornos Neurocognitivos.

[4] World Health Organization (2017). Global action lano n the public health response to dementia 2017-2025.

[5] World Alzheimer Report (2018). The state of the art of dementia research: new frontiers.

[6] McMurtray A, Saito E, Nakamoto B (2009). Language preference and development of dementia among bilingual individuals. Hawaii Med J.

[7] Kiran S, Thompson CK (2019). Neuroplasticity of language networks in aphasia: advances, updates and future challenges. Front Neurol.

[8] Reilly J, Peelle JE, Antonucci SM, Grossman M (2011). Anomia as a marker of distinct semantic memory impairments in Alzheimer's disease and semantic dementia. Neuropsychology.

[9] De Lira JO, Ortiz KZ, Campanha AC, Bertolucci PH, Minett TS (2011). Microlinguistic aspects of the oral narrative in patients with Alzheimer's disease. Int Psychogeriatr.

[10] Cera LM, Ortiz KZ, Bertolucci PH, Minett TS (2017). Phonetic and phonological aspects of speech in Alzheimer's disease. Aphasiology.

[11] Klimova B, Maresova P, Valis M, Hort J, Kuca K (2015). Alzheimer's disease and language impairments: social intervention and medical treatment. Clin Interv Aging.

[12] Klimova B, Kuca K (2016). Speech and language impairments in dementia. J Appl Biomed.

[13] Bahar-Fuchs A, Martyr A, Goh AM, Sabates J, Clare L (2019). Cognitive training for people with mild to moderate dementia. Cochrane Database Syst Rev.

[14] Fink RB, Brecher A, Schwarts MF (2002). A computer-implemented protocol for treatment of naming disorders: evaluation of clinician-guided and partially self-guided instruction. Aphasiology.

[15] Middleton EL, Schwartz MF, Rawson KA, Garevey K (2015). Test-enhanced learning versus errorless learning in aphasia rehabilitation: testing competing psychological principles. J Exp Psychol Learn Mem Cogn.

[16] Middleton EL, Schwartz MF, Rawson KA, Traut H, Verkuilen J (2016). Towards a theory of learning for naming rehabilitation: retrieval practice and spacing effects. J Speech Lang Hear Res.

[17] Barthel G, Meinzer M, Djundja D, Rockstroh B (2008). Intensive language therapy in chronic aphasia: which aspects contribute most?. Aphasiology.

[18] Kang SHK, Gollan TH, Pashler H (2013). Don't just repeat after me: retrieval practice is better than imitation for foreign vocabulary learning. Psychon Bull Rev.

[19] Kleim JA, Jones TA (2008). Principles of experience-dependent neural plasticity: implications for rehabilitation after brain damage. J Speech Lang Hear Res.

[20] Dignam JK, Rodriguez AD, Copland DA (2016). Evidence for intensive aphasia therapy: consideration of theories from neuroscience and cognitive psychology. PM R.

[21] Carretti B, Borella E, Zavagnin M, Beni R (2013). Gains in language comprehension relating to working memory training in healthy older adults. Int J Geriatr Psychiatry.

[22] Minett TS, Dean JL, Firbank M, English P, O’Brien JT (2005). Subjective memory complaints, white-matter lesions, depressive symptoms, and cognition in elderly patients. Am J Geriatr Psychiat.

[23] Jorm AF, Christensen H, Korten AE, Jacomb PA, Henderson AS (2001). Memory complaints as a precursor of memory impairment in older people: a longitudinal analysis over 7–8 years (2001). Psychol Med.

[24] Chen J-H, Lin K-P, Chen Y-C (2009). Risk factors for dementia. J Formos Med Assoc.

[25] Soares EC, Ortiz KZ (2009). Influence of schooling on language abilities of adults without linguistic disorders. São Paulo Med J.

[26] Kelly ME, Loughrey D, Lawlor B, Robertson IH, Walsh C, Brennan S (2014). The impact of cognitive training and mental stimulation on cognitive and everyday functioning of healthy older adults: a systematic review and meta-analysis. Ageing Res Rev.

[27] Jensen AR (1993). Why is reaction time correlated with psychometricg?. Curr Dir Psychol Sci.

[28] Cotelli M, Manenti R, Brambilla M, Zanetti O, Miniussi C (2012). Naming ability changes in physiological and pathological aging. Front Neurosci.

[29] McKhann GM, Knopman DS, Chertkow H, Hyman BT, Clifford RJ, Kawas CH (2011). The diagnosis of dementia due to Alzheimer's disease: recommendations from the National Institute on Aging-Alzheimer's Association workgroups on diagnostic guidelines for Alzheimer's disease. Alzheimers Dement.

[30] Engelhardt E, Tocquer C, André C, Moreira DM, Okamoto IH, Cavalcanti JL (2011). Vascular dementia: diagnostic criteria and supplementary exams. Dement Neuropsychol.

[31] Lawton MP, Brody EM (1969). Assessment of older people: self-maintaining and instrumental activities of daily living. Gerontologist.

[32] Bertolucci PHF, Brucki SMD, Campacci SR, Juliano Y (1994). The mini-mental state examination in an outpatient populational: influence of literacy. Arq Neuro-Psiquiatr.

[33] Brucki SMD, Nitrini R, Caramelli P, Bertolucci PHF, Okamoto IH (2003). Suggestions for utilization of the mini-mental state examination in Brazil. Arq Neuro-Psiquiatr.

[34] Parente MAMP, Fonseca RP, Pagliarin KC (2016). Bateria Montreal-Toulouse de Avaliação da Linguagem - Bateria MTL-Brasil.

[35] Moraes AL, Guimarães LSP, Joanette Y, Parente MA, Fonseca RP, Almeida RM (2013). Effect of aging, education, reading and writing, semantic processing and depression symptoms on verbal fluency. Psicol Reflex Crit.

[36] Pedrosa YH (2007). Normative data for standardization and determination of more viable semantic categories of verbal fluency test for Brazilian Portuguese language.

[37] Radanovic M, Mansur LL, Scaff M (2004). Normative data for the Brazilian population in the Boston Diagnostic Aphasia Examination: influence of schooling. Braz J Med Biol Res.

[38] Mansur LL, Radanovic M, Araújo GC, Taquemori LY, Greco LL (2006). Teste de nomeação de Boston: desempenho de uma população de São Paulo. Pró-Fono R Atual Cient.

[39] Brucki SM, Rocha MS (2004). Category fluency test: effects of age, gender and education on total scores, clustering and switching in Brazilian Portuguese-speaking subjects. Braz J Med Biol Res.

[40] Clark DO, Xu H, Unverzagt F, Hendrie H (2016). Does targeted cognitive training reduce educational disparities in cognitive function among cognitively normal older adults?. Int J Geriatr Psychiatry.

[41] Tardif S, Simard M (2011). Cognitive stimulation programs in healthy elderly: a review. Int J Alzheimers Dis.

[42] Byng S, Nickels L, Black M (1994). Replicating therapy for mapping deficits in agrammatism: remapping the deficit?. Aphasiology.

[43] Nickels L, Best W (1996). Therapy for naming disorders (Part II): Specifics, surprises and suggestions. Aphasiology.

[44] López-Higes R, Martín-Aragoneses MT, Rubio-Valdehita S, Delgado-Losada ML, Montejo P, Montenegro M (2018). Efficacy of cognitive training in older adults with and without subjective cognitive decline is associated with inhibition efficiency and working memory span, not with cognitive reserve. Front Aging Neurosci.

[45] Matysiak O, Kroemeke A, Brzezicka A (2019). Working memory capacity as a predictor of cognitive training efficacy in the elderly population. Front Aging Neurosci.

